# Analysis of the Characteristics of Colonoscopy Perforation and Risk Factors for Failure of Endoscopic Treatment

**DOI:** 10.7759/cureus.25677

**Published:** 2022-06-05

**Authors:** Zhi Jiehua, Ali Kashif, Che YaoSheng, Sun YunYun, Liang Lanyu

**Affiliations:** 1 Department of Gastroenterology, The Affiliated Hospital of Yangzhou University, Yangzhou University, Yangzhou, CHN; 2 Department of Gerontology, The Affiliated Hospital of Yangzhou University, Yangzhou University, Yangzhou, CHN

**Keywords:** intestinal perforation, retrospective observational study, endoscopic therapy, perforation, colonoscopy

## Abstract

Background: Many studies have been done regarding perforation after colonoscopy, but few studies analyzed the risk factors of endoscopic treatment failure after colonoscopy perforation. This study aimed to analyze the clinical characteristics and treatment plan of those patients with perforation after colonoscopy diagnosis and the treatment and risk factors of failure to endoscopic treatment.

Method: This was a retrospective observational study of patients who underwent colonoscopy examination and treatment at the Affiliated Hospital of Yangzhou University, from 04/2009 to 03/2020. The patients were grouped as perforation, treatment success, or failure (required laparoscopy or laparotomy).

Results: From April 2009 to March 2020, 43,470 patients were examined and treated with colonoscopy. There were 35 cases of intestinal perforation, for an incidence of 0.081%. Four patients had immediate surgical intervention (two patients with laparoscopic surgery and two with laparotomy surgery). Thirty-one (88.57%) patients underwent endoscopic treatment. Endoscopic treatment was successful in 20 patients and failed in 11. Compared with the failure group, the perforation size in the success group was smaller (7.60±4.85 vs. 14.4±7.03 mm, P=0.004), hospital stay was shorter (26.6±13.1 vs. 14.2±3.0, P=0.011), and hospitalization costs were lower (30,208±9506 vs. 23,053±6227 RMB, P=0.002). Multivariable logistic stepwise analysis showed that the absence of abdominal pain after therapeutic colonoscopy was independently associated with the success of endoscopic treatment.

Conclusions: Endoscopic treatment is logically the preferred modality for perforation management, leading to good recovery, shorter hospital stay, and lower costs of treatment. Postoperative abdominal pain is significantly related to the failure of endoscopic treatment.

## Introduction

Colorectal cancer (CRC) is a malignant neoplasm of the colon or rectum, and the third most common cancer worldwide [1]. It mostly affects persons >60 years of age (75% of the patients) and men are more commonly affected than women [1-2]. The lifetime risk of both colon cancer and rectal cancer is 2.71% for men and 1.77% for women [3]. The age-standardized rate of CRC incidence increased from 12.8 in 2003 to 16.8 per 100,000 in 2011, while the mortality rose from 5.9 to 7.8 per 100,000. Therefore, CRC represents an increasing threat in China [4-5].

Relevant associations in Europe and the United States explicitly stated that colonoscopy is an important method for CRC screening and management [2-7]. Nevertheless, despite its benefits in the prevention and management of CRC, colonoscopy is associated with non-negligible risks of hemorrhage and perforation, which increase the morbidity of the procedure [8-12].

With the further development of China's economy and society, the number of colonoscopies and treatments is expected to increase as well, and the number of complications, such as bleeding and perforation, are expected to increase correspondingly [13]. In 2014, the American Society of Gastroenteroscopy (ASGE) and the American Society of Gastroenterology jointly issued a statement on the quality index of colonoscopy, which suggested that the incidence of perforation during therapeutic colonoscopy should be <1/500, and the incidence of perforation during diagnostic colonoscopy should be <1/1000 [14]. Although the incidence of perforation is very low, the consequences are serious, some patients have to undergo an operation, and the condition is sometimes even life-threatening [8-12].

Many studies have been done about perforation after colonoscopy, but few studies analyzed the risk factors of endoscopic treatment failure after colonoscopy perforation. Therefore, the aim of the present study was to analyze the clinical characteristics and treatment plan of those patients with perforation after colonoscopy diagnosis and treatment in one hospital over recent years and examine the risk factors for failure of endoscopic treatment after perforation.

## Materials and methods

Study design and patients

This was a retrospective observational study of patients who underwent colonoscopy examination and treatment at the Department of Gastroenterology in an affiliated Hospital of Yangzhou University, from April 2009 to March 2020. This study was approved by the Ethics Committee of the Affiliated Hospital of Yangzhou University (approval no. 2021-YKL06-09-004). The need for individual consent was waived by the committee. 

The inclusion criteria were: 1) received diagnostic or/and therapeutic endoscopic examination; 2) intraoperative diagnosis or postoperative radiographic diagnosis of colon perforation; and 3) the perforation was treated by endoscopy. The exclusion criteria were: 1) patients with incomplete data; or 2) received surgery directly after perforation.

Data collection 

Age, sex, history of abdominal operation, hospital stay, cost of treatment, indications for diagnostic colonoscopy and therapeutic colonoscopy, quality of bowel preparation before the colonoscopy (BBPS score), size of the lesion, location of the lesion, treatment of the lesion (endoscopic treatment, abdominal surgery, or laparoscopy), 24-h abdominal pain, and temperature were collected from the medical charts.

Grouping

According to the outcome of the endoscopic treatment, the patients were allocated to the success group or the failure group. The patients in the failure group received laparoscopy or laparotomy to manage the perforation. The potential risk factors of endoscopic treatment failure were analyzed. The diagnosis of perforation was made according to the observation of peritoneal structures during colonoscopy or free intraperitoneal air which was detected by abdominal CT examination [15]. All colonoscopies were performed by associate chief physicians with an experience of >1000 cases.

Statistical analysis

SPSS 23.0 (IBM Corp., Armonk, NY) was used for statistical analysis. Continuous data were tested for normal distribution using the Kolmogorov-Smirnov test. Normally distributed continuous data are shown as means ± standard deviation and were analyzed using the Student t-test. Continuous data with a skewed distribution were presented as medians (range) and were analyzed using the Mann-Whitney U test. The differences between rates were tested by χ2 or Fisher exact tests, when appropriate. Multivariable logistic stepwise regression was used to analyze the potential independent risk factors for failure of endoscopic treatment of colonoscopy perforation. P values <0.05 were considered statistically significant.

## Results

Characteristics of the patients

From April 2009 to March 2020, 43,470 patients received examination and treatment by colonoscopy. A total of 35 incidents (0.081%) of colonoscopic perforation were reported (16 males and 19 females), of which 11 cases occurred during diagnostic colonoscopy and 24 cases after therapeutic colonoscopy (three polypectomy cases, five endoscopic mucosal resections, and 16 endoscopic mucosal dissections). Incidents of colonoscopic perforation are 0.029%% and 0.426% for diagnostic and therapeutic colonoscopy, respectively. The age ranged from 39 to 82 years (on average, 58 years). The location of perforation was in the rectum (n=7), sigmoid colon (n=18), descending colon (n=3), transverse colon (n=3), ascending colon (n=2), and ileocecum (n=2). Thirty-two patients (91. 4%) were discovered within 24 hours (<24h).

Perforation management

Four patients had immediate surgical intervention (two patients with laparoscopic surgery and two with laparotomy surgery). Thirty-one (88.57%) patients underwent endoscopic treatment. Endoscopic treatment was successful in 20 patients and failed in 11. Eight patients in the failure group received laparoscopic treatment, and three were treated by laparotomy. Abrosia, anti-infection, and nutritional support were given after the operation. All patients recovered after treatment. The clinical data of the two groups are shown in Table 1.

**Table 1:** Characteristics of patients who underwent endoscopic treatment

Variable	Success group (n=20)	Failure group (n=11)	P-value
Sex, n (%)			0.258
Male	11 (55.0)	3 (27.3)	
Female	9 (45)	8 (72.7)	
Age (years)	65.4±10.8	63.6±11.3	0.680
Preoperative body temperature (ºC)	36.8±0.2	36.8±0.1	0.903
Preoperative leukocytes (10^9^/L)	6.70±0.86	6.85±0.69	0.623
Body mass index (kg/m^2^)	21.7±2.1	22.3±2.8	0.526
Hospital stay (days)	14.2±3.0	26.6±13.1	0.011
Hospitalization costs (RMB)	23,054±6227	30,209±9507	0.017
Perforation size (mm)	7.6±4.9	14.4±7.0	0.004
The Boston bowel preparation scale	8.5±0.6	7.9±0.5	0.020

**Figure 1:**
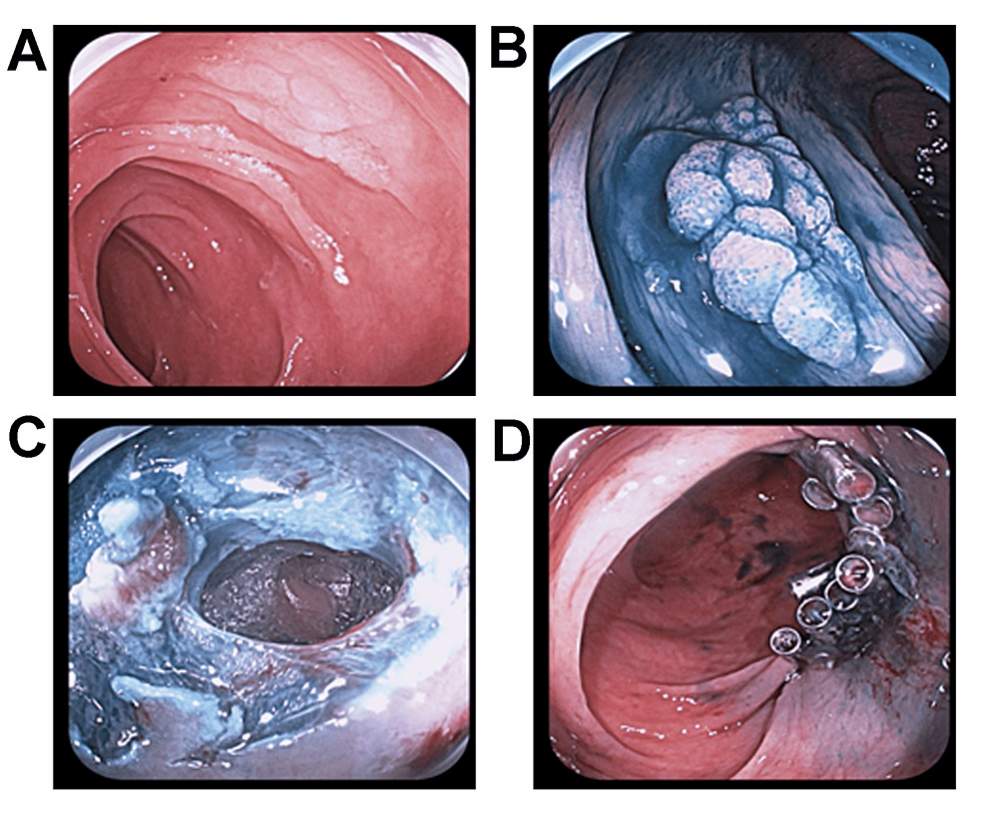
Figure 1. A typical case of perforation during endoscopic treatment. (A) Prior to lesion staining. (B) The lesion was stained with a 0.2% indigo rouge solution. (C) Perforation was detected intraoperatively. (D) Titanium clips close the wound after perforation.

There were no significant differences in age, sex, body mass index (BMI), preoperative temperature, leukocyte, and abdominal surgery history between the two groups (all P>0.05). The quality of intestinal preparation in the success group was significantly better than that in the failure group (P=0.02). Compared with the failure group, the perforation size in the success group was smaller (7.60±4.85 vs. 14.4±7.03 mm, P=0.004), hospital stay was shorter (26.6±13.1 vs. 14.2±3.0, P=0.011), and hospitalization costs were lower (30,208±9506 vs. 23,053±6227 RMB, P=0.002) (shown in Table 1). Figure 1 presents a typical case of perforation successfully treated by endoscopy.

Univariable and multivariable analyses

Univariable logistic regression analyses showed that therapeutic colonoscopy and perforation <15 mm were associated with the success of endoscopic therapy. Compared with the success group, patients in the failure group had more abdominal pain, fever, and elevated neutrophils 24 hours postoperatively (Table 2). These signs suggest that patients with intestinal perforation should be closely observed for postoperative signs and symptoms such as pain, fever, and abnormal blood routine. 

**Table 2:** Univariable analyses of endoscopic treatment success

Variables	Success group (n=20)	Failure group (n=11)	P-value
Indications, n (%)			0.001
Diagnostic	1 (5.0)	7 (63.6)	
Treatment	19 (95.0)	4 (36.4)	
History of abdominal operation, n (%)	9 (45.0)	4 (36.4)	0.078
Diameter of perforation, n (%)			
>15 mm	5 (25.0)	8 (72.7)	0.021
<15 mm	15 (75.0)	3 (27.3)	
Location of perforation, n (%)			0.51
Proximal colon	6 (30.0)	2 (18.2)	
Colon sigmoid	8 (40.0)	9 (81.8)	
Rectum	6 (30.0)	0	
Postoperative abdominal pain, n (%)	3 (15.0)	10 (90.9)	<0.001
Postoperative body temperature, n (%)			0.006
Beyond normal range	4 (20.0)	8 (72.7)	
Over normal range	16 (80.0)	3 (27.3)	
Postoperative leukocyte, n (%)			0.007
Beyond normal range	5 (25.0)	9 (81.8)	
Over normal range	15 (75.0)	2 (18.2)	
Postoperative neutrophils, n (%)			0.020
Beyond normal range	9 (45.0)	10 (90.9)	
Over normal range	11 (55.0)	1 (9.1)	

Multivariable logistic stepwise analysis showed that the absence of abdominal pain after therapeutic colonoscopy was independently associated with the success of endoscopic treatment (Table 3).

**Table 3:** Multivariable logistic stepwise regression analysis of the success of endoscopic treatment a. Variable(s) entered on step 1: Postoperative abdominal pain. b. Variable(s) entered on step 2: Physical signs

Variables		P-value	odd ratio	95% Confidence Interval	
				Lower	Upper
Step 1^a^	Postoperative abdominal pain	0.001	0.018	0.002	0.193
	Constant	0.002	188.889		
Step 2^b^	Physical signs	0.075	0.069	0.004	1.304
	Postoperative abdominal pain	0.008	0.031	0.002	0.409
	Constant	0.007	8666.232		

## Discussion

Many studies have reported on perforation after colonoscopy [8-12], but few studies analyzed the risk factors of endoscopic treatment failure after colonoscopy perforation. Therefore, the present study aimed to analyze the clinical characteristics and treatment plan of those patients with perforation after colonoscopy diagnosis and treatment. The results suggest that endoscopic treatment is an alternative treatment modality to conservative or surgical management for perforation management, leading to good recovery, shorter hospital stay, and lower costs of treatment. Postoperative abdominal pain is significantly related to the failure of endoscopic treatment.

Perforation is considered one of the most serious adverse events of colonoscopy, and nearly 5% of colonoscopy perforations are fatal [8-12]. The incidence of colonoscopy perforation varies among countries and hospitals. A study in China reported an incidence of perforation of 0.012% among 110,785 cases of enteroscopy from January 2000 to December 2012 [16]. On the other hand, the incidence of perforation in diagnostic and therapeutic colonoscopy is 0.02%-0.8% and 0.02%-3%, respectively, in western countries [17]. In the past decade, the incidence of perforation at our center was 0.081%. This incidence is lower than the requirements of the American Society of Gastroenteroscopy (ASGE) and the American Society of Gastroenterology joint statement for colonoscopy, recommencing an overall incidence of perforation of <0.1% for diagnostic colonoscopy and <0.05% for screening colonoscopy. In this study, most perforations occurred in the rectum sigmoid colon (73.0%), which was consistent with a previous study (50%-88%) [14].

Treatment of endoscopic perforation includes conservative treatment, endoscopic treatment, and surgical treatment [18]. With the development of endoscopic equipment and the increase in doctors’ skills in endoscopic operation, endoscopic treatment of intestinal perforation has been increasingly reported [19-20]. Perforations <1 cm can be effectively cured under therapeutic colonoscopy, with success rates of 60%-90% [21]. Compared with surgical operations, hospital stays are shorter and costs are smaller [22].

Although surgical management could provide definitive treatment for patients with endoscopic perforation, when the risk associated with general anesthesia, postoperative complications, legal problems, and hospital costs are taken into account, endoscopic management is logically the preferred modality. On the other hand, a failure in endoscopic management could result in delayed treatment, thereby causing morbidity and mortality. Therefore, it is essential for us to accurately determine the factors that lead to endoscopic failure to treat in a timely manner. Studies have shown that perforation >15 mm was the only predictor of endoscopic treatment failure [23]. Our study found that therapeutic colonoscopy, perforation <15 mm, and the absence of abdominal pain were associated with the success of endoscopic therapy. Fever, elevated neutrophils, and severe abdominal pain after endoscopic treatment within 24h indicated that endoscopic treatment failed and that surgery was required. Multivariable logistic stepwise analysis showed that the absence of abdominal pain after therapeutic colonoscopy was independently associated with the success of endoscopic treatment.

For patients receiving endoscopic treatment, close observation for postoperative symptoms and signs is needed to detect abdominal pain and abdominal distension. Laparoscopy or laparotomy should be done immediately to ensure optimal outcomes. Experience and training in how to close the perforation might be the keys to success.

A recent study on endoscopic tubing drainage is promising for rescuing endoscopy-associated perforation [24]. A colonic transendoscopic enteral tube (TET) (outer diameter 2.7 mm) with loops can be fixed onto the colon wall close to the perforation site by endoscopic clips. The timely drainage using the colonic TET was reported as the core management approach to avoid surgery in patients with an endoscopy-associated perforation. However, the colonic TET technique was not used in the current selection population in our center.

Our study may be important because of 3 important clinical implications. First, it reflects a real-life experience on the feasibility of endoscopic closure of colon perforations occurring during consecutive diagnostic or therapeutic colonoscopies. Second, it is most important to observe the symptoms and signs of patients after the endoscopic closure of colon perforations. Our research analysis showed that the absence of abdominal pain was independently associated with endoscopic treatment success. Last, our data show the current areas with potential room for improvement, such as increasing the endoscopic skills to solve an iatrogenic colon injury and attempt closure of larger defects.

This study has limitations. This study was a single-center retrospective study. Due to the low incidence of colonoscopy perforation, a small number of cases of perforation were included and a selection bias is likely. Second, for the examination of the factors associated with success, we were limited to the variables that could be found in the medical charts. Prospective multicenter studies are needed to confirm the relative information on complications of colonoscopy.

## Conclusions

In conclusion, we suggest that endoscopic treatment could be a choice for iatrogenic gastrointestinal perforation, but the indications should be strictly controlled. For patients with perforation, perforation size >1.5cm, poor intestinal preparation quality, and postoperative symptoms and signs should suggest close monitoring. The management decision on the endoscopy associated perforation will be improved mainly based on the related endoscopic technique development.

## References

[1] Kuipers EJ, Grady WM, Lieberman D (2015). Colorectal cancer. Nat Rev Dis Primers.

[2] Glynne-Jones R, Wyrwicz L, Tiret E, Brown G, Rödel C, Cervantes A, Arnold D (2017). Rectal cancer: ESMO Clinical Practice Guidelines for diagnosis, treatment and follow-up. Ann Oncol.

[3] Bray F, Ferlay J, Soerjomataram I, Siegel RL, Torre LA, Jemal A (2018). Global cancer statistics 2018: GLOBOCAN estimates of incidence and mortality worldwide for 36 cancers in 185 countries. CA Cancer J Clin.

[4] Zhu J, Tan Z, Hollis-Hansen K, Zhang Y, Yu C, Li Y (2017). Epidemiological trends in colorectal cancer in China: an ecological study. Dig Dis Sci.

[5] Guo T, Xie L, Zhao J (2018). Trend analysis of morbidity and mortality of colorectal cancer in China from 1988 to 2009 (Article in Chinese). Zhonghua Wei Chang Wai Ke Za Zhi.

[6] Bibbins-Domingo K, Grossman DC, Curry SJ (2016). Screening for colorectal cancer: US preventive services task force recommendation statement. JAMA.

[7] von Karsa L, Patnick J, Segnan N (2013). European guidelines for quality assurance in colorectal cancer screening and diagnosis: overview and introduction to the full supplement publication. Endoscopy.

[8] Arana-Arri E, Imaz-Ayo N, Fernández MJ (2018). Screening colonoscopy and risk of adverse events among individuals undergoing fecal immunochemical testing in a population-based program: a nested case-control study. United European Gastroenterol J.

[9] Fisher DA, Maple JT, Ben-Menachem T (2011). Complications of colonoscopy. Gastrointest Endosc.

[10] Dulskas A, Smolskas E, Kildusiene I (2019). Outcomes of surgical management of iatrogenic colonic perforation by colonoscopy and risk factors for worse outcome. J Buon.

[11] Castro G, Azrak MF, Seeff LC, Royalty J (2013). Outpatient colonoscopy complications in the CDC's Colorectal Cancer Screening Demonstration Program: a prospective analysis. Cancer.

[12] Kim SY, Kim HS, Park HJ (2019). Adverse events related to colonoscopy: global trends and future challenges. World J Gastroenterol.

[13] Li D (2018). Recent advances in colorectal cancer screening. Chronic Dis Transl Med.

[14] Rex DK, Schoenfeld PS, Cohen J (2015). Quality indicators for colonoscopy. Am J Gastroenterol.

[15] Levy I, Gralnek IM (2016). Complications of diagnostic colonoscopy, upper endoscopy, and enteroscopy. Best Pract Res Clin Gastroenterol.

[16] Shi X, Shan Y, Yu E (2014). Lower rate of colonoscopic perforation: 110,785 patients of colonoscopy performed by colorectal surgeons in a large teaching hospital in China. Surg Endosc.

[17] Martínez-Pérez A, de'Angelis N, Brunetti F (2017). Laparoscopic vs. open surgery for the treatment of iatrogenic colonoscopic perforations: a systematic review and meta-analysis. World J Emerg Surg.

[18] Yang DH, Byeon JS, Lee KH (2010). Is endoscopic closure with clips effective for both diagnostic and therapeutic colonoscopy-associated bowel perforation?. Surg Endosc.

[19] de'Angelis N, Di Saverio S, Chiara O (2018). 2017 WSES guidelines for the management of iatrogenic colonoscopy perforation. World J Emerg Surg.

[20] Park JY, Choi PW, Jung SM, Kim NH (2016). The outcomes of management for colonoscopic perforation: a 12-Year experience at a single institute. Ann Coloproctol.

[21] Thirumurthi S, Raju GS (2015). Management of polypectomy complications. Gastrointest Endosc Clin N Am.

[22] Kantsevoy SV, Bitner M, Hajiyeva G (2016). Endoscopic management of colonic perforations: clips versus suturing closure (with videos). Gastrointest Endosc.

[23] An SB, Shin DW, Kim JY, Park SG, Lee BH, Kim JW (2016). Decision-making in the management of colonoscopic perforation: a multicentre retrospective study. Surg Endosc.

[24] Zhang F, Wen Q, Cui B (2022). Drainage via colonic transendoscopic enteral tubing increases our confidence in rescuing endoscopy-associated perforation. Endoscopy.

